# Unraveling the interaction between pathological upper limb synergies and compensatory trunk movements during reach-to-grasp after stroke: a cross-sectional study

**DOI:** 10.1007/s00221-012-3169-6

**Published:** 2012-07-12

**Authors:** Joost van Kordelaar, Erwin E. H. van Wegen, Gert Kwakkel

**Affiliations:** 1 Department of Rehabilitation Medicine, MOVE Research Institute Amsterdam, VU University Medical Center, De Boelelaan 1117, 1081 HV Amsterdam, The Netherlands; 2 Department of Rehabilitation Medicine, Rudolf Magnus Institute of Neuroscience, University Medical Center Utrecht, Utrecht, The Netherlands

**Keywords:** Stroke, Synergy, Compensation, Kinematics, Upper limb, Motor control

## Abstract

The aim of the present study was to identify how pathological limb synergies between shoulder and elbow movements interact with compensatory trunk movements during a functional movement with the paretic upper limb after stroke. 3D kinematic joint and trunk angles were measured during a reach-to-grasp movement in 46 patients with stroke and 12 healthy individuals. We used principal component analyses (PCA) to identify components representing linear relations between the degrees of freedom of the upper limb and trunk across patients with stroke and healthy participants. Using multivariate logistic regression analysis, we investigated whether component scores were related to the presence or absence of basic limb synergies as indicated by the arm section of the Fugl-Meyer motor assessment (FMA). Four and three principal components were extracted in patients with stroke and healthy individuals, respectively. Visual inspection revealed that the contribution of joint and trunk angles to each component differed substantially between groups. The presence of the flexion synergy (Shoulder Abduction and Elbow Flexion) was reflected by component 1, whereas the compensatory role of trunk movements for lack of shoulder and elbow movements was reflected by components 2 and 3 respectively. The presence or absence of basic limb synergies as determined by means of the FMA was significantly related to components 2 (*p* = 0.014) and 3 (*p* = 0.003) in patients with stroke. These significant relations indicate that PCA is a useful tool to identify clinically meaningful interactions between compensatory trunk movements and pathological synergies in the elbow and shoulder during reach-to-grasp after stroke.

## Introduction

Recovery of upper limb function after stroke typically evolves in a rather predictable pattern that has been explicitly described by Twitchell (1951). He noted a remarkable uniformity in the manner and sequence in which basic limb synergies emerged before isolated movements of the various joints could be mastered. These basic limb synergies involve pathological couplings between shoulder and elbow movements, which are the result of increased co-activation between muscles in the paretic upper limb that can be elicited voluntarily or as a reflexive reaction (Twitchell 1951). As a consequence, the joints that are coupled within a synergy cannot be mastered in isolation. In patients with stroke, two basic limb synergies can be distinguished for the paretic upper limb, viz. (1) abduction and external rotation of the shoulder, flexion of the elbow, and supination of the forearm when elevating the paretic arm (i.e., flexion synergy); and (2) adduction and internal rotation of the shoulder, extension of the elbow, and pronation of the forearm when stretching the elbow (i.e., extension synergy) (Brunnstrom 1970).

Based on Twitchell’s (Twitchell 1951) observations, several authors have explicitly defined the stages in which motor recovery of the paretic upper limb evolves after stroke and have developed several clinical assessments to determine the stage of recovery in patients with stroke (Brunnstrom 1970; Fugl-Meyer et al. 1975; Gowland 1990). One of these assessments is the Fugl-Meyer motor assessment (FMA) of the paretic arm, which is a valid and reliable clinical assessment (Sanford et al. 1993) to quantify the ability of patients with stroke to perform dissociated (i.e., out-of-synergy) arm movements (Fugl-Meyer et al. 1975).

The exact pathophysiological mechanisms that underly these basis limb synergies and velocity-dependent exaggeration of myotatic reflexes (i.e., spasticity), as quantified by the FMA, are still unclear. However, several hypotheses have been postulated (Gracies 2005). For instance, there are indications that exaggerated responses to tonic and phasic muscle stretch are caused by reduced descending inhibitory control onto mainly Ia afferents (Aymard et al. 2000; Faist et al. 1994). In addition, increased co-contraction of various muscles in the paretic limb may be caused by reduced recurrent inhibition of Renshaw cells onto alpha motor neurons that control voluntary movements (Katz and Pierrot-Deseilligny 1982), although the role of recurrent inhibition for complex upper limb motor tasks remains unclear (Katz and Pierrot-Deseilligny 1999). Based on these hypothesized neurological mechanisms, reductions of pathological synergies are often seen as a reflection of “true neurological repair” (Kwakkel et al. 2004; Prabhakaran et al. 2008; Zarahn et al. 2011).

However, the concept of a “synergy” is not only used to indicate the severity of motor impairments. Regarding general principles of motor control, it has been argued that a synergy comprises a functional linkage between joints and/or muscles that is used by the motor system to reduce the number of degrees of freedom (Bernstein 1967) involved in a particular task (Turvey 1990). The joints that are involved in a functional linkage or synergy (Turvey 1990) thus share a common coordination pattern that is adopted to execute a functional task, such as reaching (Latash et al. 2003).

With respect to reaching, there is ample evidence that these functional linkages or synergies are changed in patients with stroke. For example, the relative timing of shoulder and elbow movements (i.e., interjoint coordination) is disrupted in patients with stroke and depends on the direction in which the hand has to be moved (Levin 1996). In addition, compared with healthy adults, particularly the contribution of the elbow is reduced, whereas increased contribution of trunk movements is typically observed (Roby-Brami et al. 2003). Furthermore, by using principal component analysis, Reisman and Scholz (2003) showed that patients with stroke use fewer joint combinations during pointing movements as compared to healthy subjects. These observations support the hypothesis that the pathological couplings between the shoulder and elbow reduce the number of degrees of freedom in the paretic upper limb that can be used during reaching and that trunk movements are used to compensate for this reduction in degrees of freedom (Levin et al. 2009). In contrast to the upper limb, which is predominantly innervated by contralateral corticospinal pathways (Palmer and Ashby 1992), trunk muscles receive extensive bilateral input from corticospinal pathways (Ferbert et al. 1992; Schwerin et al. 2008). While contralateral pathways may be severely damaged after stroke, trunk muscles may still depend on intact ipsilateral pathways, which might explain the tendency of patients with stroke to employ trunk movements as a strategy to compensate for upper limb impairments.

However, pathological upper limb synergies and compensatory trunk movements constitute a complex interaction during reaching movements. Kinematically, the trunk, shoulder, and elbow form a chain of 8 degrees of freedom, yielding a linked segment system that can potentially adapt in innumerable ways to motor impairments such as pathological synergies in the paretic upper limb. Unfortunately, it is still largely unclear how patients with stroke employ the degrees of freedom of the paretic upper limb and trunk and how these degrees of freedom are correlated. As a consequence, it remains unclear whether changes in the recruited brain areas as observed in fMRI studies contribute to restitution of motor control rather than adaptive motor control (Buma et al. 2010).

In the present study, we investigated how compensating trunk movements and pathological joint synergies in the paretic upper limb interact within functional synergies during a reach-to-grasp task. Previous studies have indicated that the pathological coupling between abduction of the shoulder and flexion of the elbow may limit forward reaching distance of the paretic upper limb (Ellis et al. 2008), suggesting that pathological synergies and compensating trunk movements become more prominent as the hand moves forward. Therefore, we used the moment in a reach-to-grasp paradigm where the hand is in the most forwardly located position to measure trunk, shoulder, and elbow angles. The aim of the study was threefold. First, we investigated differences in trunk, shoulder, and elbow angles between patients with stroke and healthy subjects. Second, by using PCA, we aimed to identify principal components that represented linear relations between the degrees of freedom of the elbow, shoulder, and trunk across patients with stroke during a reach-to-grasp task. A control group of healthy individuals was used to assess whether the identified components were typical of patients with stroke. Third, by using multivariate logistic regression analysis, we investigated whether the identified components were associated with the presence or absence of basic limb synergies as assessed by the FMA.

## Methods

### Participants

Forty-eight patients with stroke were included in the present study. However, the data of two patients could not be used due to errors in the data from the anatomical calibration. This resulted in a sample of forty-six patients. In addition, twelve healthy participants were measured, with no reported history of neurological and/or orthopedic disorders. Participant characteristics are presented in Table 1. Stroke was defined according to the World Health Organization criteria (Hatano 1976). Type and localization of stroke were determined using CT or MRI scans. Patients who met the following criteria were included: (1) having experienced a first-ever ischemic or hemorrhagic stroke involving the territory of the medial or anterior cerebral artery as revealed by computerized axial tomography or magnetic resonance imaging scan; (2) aged between 18 and 80 years; (3) able to sit without trunk support for at least 30 s; (4) showing motor deficits in the arm and/or hand, but nevertheless able to grasp objects; (5) no severe deficits in memory and understanding as indicated by a score of 23 or higher on the mini mental state examination (MMSE); (6) no severe deficits in communication as indicated by a score of 5 on the Utrecht Communication Observation (UCO); (7) no complicating medical history such as cardiac, pulmonary, or orthopedic disorders; (8) having provided written informed consent and having sufficient motivation to participate.

**Table 1:** Participant characteristics

Characteristic	Total
Patients with stroke	
N	46
Gender, F/M	15/31
Mean age (SD), years	60.30 (12.59)
Paretic body side, L/R	22/24
Mean time interval (weeks) between stroke and measurement^a^	26 (3–447)
Kind of stroke, hemorrhagic/ischemic	1/45
Type of stroke (Bamford)	
LACI	32
PACI	13
TACI	1
NIHSS b	1 (0–4)
ARAT total score^b^	45 (38–57)
FMA upper limb (0–66)^b^	63 (51–65)
FMA arm (0–36)^b^	35 (29–36)
FMA wrist (0–10)^b^	10 (7–10)
FMA hand (0–14)^b^	14 (13–14)
FMA upper limb coordination (0–6)^b^	5 (4–6)
Healthy subjects	
N	12
Gender, F/M	5/7
Mean age (SD), years	52.75 (5.88)

*ARAT* action research arm test, *F* female, *FMA* Fugl-Meyer motor assessment, *L* left, *LACI* lacunar anterior cerebral infarction, *M* male, *N* number of subjects, *NIHSS* National Institutes of Health Stroke Scale, *PACI* partial anterior cerebral infarction, *R* right, *TACI* total anterior cerebral infarction

^a^Median value (minimum value–maximum value)

^b^Median value (interquartile range)

The study was approved by the local Ethics Committee and was part of the EXPLICIT-stroke program, which is registered at the Netherlands National Trial Register (NTR1424). EXPLICIT-stroke is a multicenter translational research program, which aims to investigate the mechanisms of recovery and the effects of early applied intensive intervention on regaining dexterity after stroke (Kwakkel et al. 2008).

### Clinical evaluation

Prior to each measurement, several clinical assessments were conducted in the patients with stroke. The National Institutes of Health Stroke Scale (NIHSS) was used to assess the severity of the lesion. The action research arm test (ARAT) was used to quantify the ability to perform functional tasks with the paretic upper limb. The upper extremity section of the Fugl-Meyer motor assessment (FMA) was used to detect the presence of basic limb synergies in the upper limb and to assess hand function.

### Kinematic data collection

**Fig. 1:**
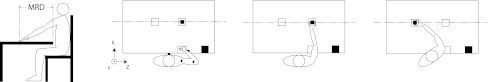
Determination of maximum reaching distance (see text) and task execution. Subject starts in the initial position (*left*). Subject reaches for the block (*small black square*) at the block position (*middle*) and places the block at the end position (*right*). The magnetic source is represented by the *large black square*. The *small rectangles* on the subject (*left*) indicate the position of the sensors. The *dashed line* represents the maximum reaching distance of the arm (*MRD*)

Kinematic data of the trunk, scapula, upper arm, and forearm were recorded by means of a portable 6 degrees of freedom electromagnetic tracking device (Polhemus Liberty, Polhemus, Vermont, USA). All movements were measured relative to a global reference frame with its origin at the center of the magnetic source, *x*-axis directed forward, *y*-axis directed upward, and *z*-axis directed rightward (Fig. 1). The sampling frequency was 240 Hz.

The motion sensors were attached to the thorax, scapula, upper arm, and lower arm using double-sided adhesive tape (Fig. 1). In patients with stroke, sensors were attached to the paretic arm, whereas in the healthy participants, sensors were attached to the non-dominant arm. An anatomical calibration procedure was carried out before each measurement, which involved digitizing the position of each of 13 anatomical landmarks relative to the global reference frame, using a pointer device or stylus (ST8, Polhemus). The position of each landmark was subsequently rotated from the global reference frame into the local reference frame of its associated sensor. In addition, the location of the gleno-humeral joint was calculated using linear regression from the scapular landmarks (Meskers et al. 1998). A list of anatomical landmarks and the mathematical calculations to construct the segment reference frames for the trunk, upper arm, and forearm are provided in the “Appendix.”

### Procedure

**Fig. 2:**
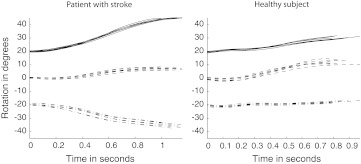
Time series of trunk rotations during seven reach-to-grasp movements from start of reach-to-grasp to end of reach-to-grasp, obtained from a patient with stroke (*left*) and a healthy individual (*right*). The curves represent Forward Trunk Rotation (*solid*), Lateral Trunk Rotation (*dash-dot*), and Longitudinal Trunk Rotation (*dashed*). An offset of +20 and −20° was added to Forward Trunk Rotation and Lateral Trunk Rotation, respectively, to better distinguish the curves. The *graphs* indicate that trunk rotations were largest at the end of the reach-to-grasp movement

While seated behind a table with a height of 76 cm, participants performed a functional movement with the affected arm that consisted of two parts, viz. (1) a reach-to-grasp movement toward a block, followed by (2) a displacement of the block toward a target location. The reach-to-grasp movement started with the hand in the initial hand position (IP), which was in front of the shoulder on the edge of the table and with the thumb against the index finger. Participants were asked to grasp and displace a block at their preferred speed after the experimenter gave a verbal “GO” signal. The position of the block (BP) that had to be grasped was dependent on each participant’s individual maximum reaching distance (MRD). MRD was determined prior to each measurement by instructing the participant to reach forward as far as possible and touch the table with the non-paretic arm while keeping the trunk against the backrest of the chair. The distance between the index finger of the non-paretic arm and the edge of the table was then used as MRD (Fig. 1). BP was located in front of the shoulder of the paretic arm at MRD. This way, the block could be grasped with minimal trunk contribution (Fig. 2), if participants had the ability to use the shoulder and to exploit the full range of motion of the elbow in the paretic arm.

The reach-to-grasp movement ended when the block was grasped and lost contact with the table. Directly after the reach-to-grasp movement, the second part of the movement started, during which the block had to be displaced toward a target position (TP), which was located at the contralateral side at a distance equal to MRD (Fig. 1).

Participants were specifically asked to grasp the block between their thumb and index finger and not to slide their hand over the table but to move it through the air. After the “GO” signal, subjects were allowed to move their trunk away from the back of the chair if this was more comfortable; however, participants were specifically instructed to remain seated and not to slide or twist over the seat of the chair throughout all motion recordings. The cubic block was 5 × 5 × 5 cm and weighed 150 g. The task was repeated until seven successful trials had been recorded.

### Data analysis

The present study focuses on the first part of the experimental paradigm: the reach-to-grasp movement. Reach-to-grasp speed profiles are characterized by a bell-shaped curve in which the maximum hand speed occurs early in the reach-to-grasp movement and gradually decreases to (almost) 0 m/s at the moment of grasping (van Vliet and Sheridan 2007). In the present study, start of reach-to-grasp was defined as the moment at which the forearm sensor exceeded 5 % of the maximum speed during the forward reach. To determine the stop threshold value, the 5 % value of the maximum hand speed during the displacement of the block was determined. This value was subsequently added to the minimum hand speed between reach-to-grasp and displacement, to obtain the stop threshold value. End of reach-to-grasp was defined as the moment at which the hand exceeded this threshold value. Movement duration was defined as the time between start of reach-to-grasp and end of reach-to-grasp.

Trunk, shoulder, and elbow rotations were calculated according to the recommendations of the International Society of Biomechanics (ISB) (Wu et al. 2005). The mathematical calculations that were used to derive the trunk, shoulder, and elbow rotations are provided in the “Appendix.” Here, trunk rotations should in fact be interpreted as combined trunk and pelvis rotations, since a pelvic sensor was not included in the experimental setup. To maintain readability, these combined pelvis and trunk rotations will be referred to as trunk rotations throughout the rest of the article.

The most forwardly located position of the hand in the present reach-to-grasp paradigm was at end of reach-to-grasp. Preliminary analysis revealed that trunk movements were indeed largest at this point (Fig. 2). Therefore, the instantaneous values of the different joint rotations at end of reach-to-grasp were included as input for the PCA. For each joint and trunk angle, the mean of the seven repetitions within each measurement was used for further analysis.

### Group differences

Independent sample *t* tests were performed to assess differences between healthy subjects and patients with stroke with respect to movement duration and trunk, shoulder, and elbow angles. The two-tailed tested significance level was set at *p* ≤ .05. Since nine separate *t* tests were used for movement duration and each of the angles, a Bonferroni correction was applied in order to avoid type I errors. This resulted in a corrected significance level of *p* ≤ .05/9 = 0.006.

### Principal component analysis

A principal component analysis (PCA) was conducted for each group separately to identify components that explained most of the variance in joint angles. Components were selected according to Kaiser’s criterion, that is, only components with an eigenvalue larger than 1 were extracted. Component rotation (Varimax) was used to maximize the dispersion of loadings within each component, to improve the interpretation of the results. Visual inspection of the component loadings was used to identify dominant joint angle contributors within each component. The individual score on each identified component (i.e., component score) was determined for each patient with stroke using the regression method.

### Multivariate logistic regression analysis

The dominance of the pathological basic limb synergies in the paretic arm was assessed with the arm section of the Fugl-Meyer motor assessment (FMA, the maximum score being 36). Since patients who are able to make complete out-of-synergy movements with the shoulder and elbow attain 34 points or higher, the FMA score was dichotomized as follows: a score of 1 was allocated to each patient who scored 34 points or higher, while a score of 0 was allocated to each patient who scored <34 points on the FMA of the paretic arm.

Multivariate logistic regression analysis was used to investigate whether the ability to make complete out-of-synergy movements (i.e., FMA ≥34) can be predicted on the basis of the component scores extracted from the principal component analysis. The component scores were inserted in the model with forced entry. The relation between observed and predicted values of the dichotomized FMA score was assessed on the basis of a 2-way contingency table, sensitivity and specificity, and positive (PPV) and negative predictive values (NPV) including their 95 % confidence intervals (95 % CI). The odds ratios of each component, including their 95 % CI, were used to assess the contribution of each component to the model. All statistical analyses were conducted using SPSS version 16.0 for Windows.

## Results

The mean movement duration was shorter for the healthy participants compared with the patients with stroke (1.10 s ± 0.24 s and 1.93 s ± 1.48 s respectively, *p* = 0.001).

**Fig. 3:**
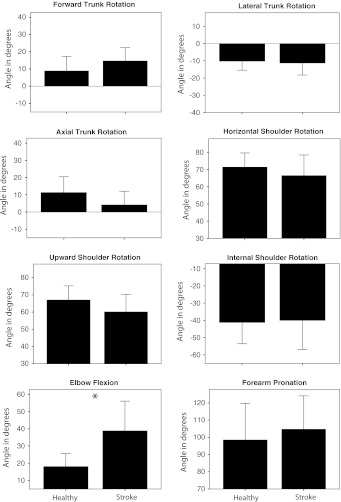
Mean joint and trunk angles for healthy subjects and patients with stroke at end of reach-to-grasp. *Error bars* represent 1 SD. An *asterisk* indicates a significant difference

The mean joint angles at the end of the reach-to-grasp movement for the patients with stroke and the healthy participants are presented in Fig. 3. Independent sample *t* tests with a Bonferroni correction for multiple testing revealed that only Elbow Flexion was significantly larger in patients with stroke as compared to healthy subjects (*t* = −3.94, *p* = 0.001).

### Principal component analysis

**Fig. 4:**
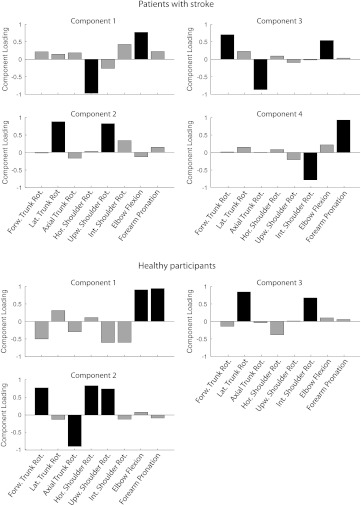
Principal components in patients with stroke and healthy participants. Positive/negative component loadings indicate a positive/negative correlation between a variable and a component. Based on visual inspection, dominant joint angle contributors (*black*) were selected within each component

For the group of patients with stroke, four principal components that had an eigenvalue larger than 1 could be extracted. The amount of variance explained by these components was 84.6 % of the total variance. By contrast, three principal components with an eigenvalue larger than 1 could be extracted in the group with healthy participants and explained 86.6 % of the total variance in this group. Figure 4 presents the loadings of each joint rotation to each component in each group. Visual inspection was used to identify the primary contributors to each component (black bars in Fig. 4).

In the patients with stroke, the primary contributors to component 1 are Horizontal Shoulder Rotation and Elbow Flexion. For component 2, the primary contributors are Lateral Trunk Rotation and Upward Shoulder Rotation. For component 3, the primary contributors are Forward Trunk Rotation, Axial Trunk Rotation, and Elbow Flexion. For component 4, the primary contributors are External Shoulder Rotation and Forearm Pronation. In the group of healthy participants, the primary contributors to component 1 were Elbow Flexion and Forearm Pronation. For component 2, the primary contributors were Forward Trunk Rotation, Axial Trunk Rotation, Horizontal Shoulder Rotation, and Upward Shoulder Rotation. For component 3, the primary contributors were Lateral Trunk Rotation and Internal Shoulder Rotation.

### Multivariate logistic regression analysis

The scores for each patient on each of the four extracted components were used in a multivariate logistic regression analysis with the dichotomized FMA score as the dependent variable. Table 2 shows the 2 × 2 contingency table, the sensitivity and specificity, and the negative (NPV) and positive (PPV) predictive values, including their 95 % CI. The presence or absence of basic limb synergies (i.e., FMA ≥34 or FMA <34, respectively) was correctly predicted by the model for 38 of the 46 included patients (82.6 %).

**Table 2:** 2 × 2 contingency table, sensitivity and specificity, negative and positive predictive values

Observed	Predicted	
	Incomplete out-of-synergy movements	Complete out-of-synergy movements
Incomplete out-of-synergy movements	14	5
Complete out-of-synergy movements	3	24
Specificity (95 % CI):	0.74 (0.55–0.85)	
Sensitivity (95 % CI):	0.89 (0.76–0.97)	
NPV (95 % CI):	0.82 (0.62–0.95)	
PPV (95 % CI):	0.83 (0.71–0.90)	

Incomplete out-of-synergy movements were defined as FMA <34; complete out-of-synergy movements were defined as FMA ≥34

*NPV* negative predictive value, *PPV* positive predictive value

Table 3 shows the beta values, odds ratios, and significance of each component for the accuracy of the model. The beta values show that components 1, 3, and 4 have a negative relation, whereas component 2 has a positive relation with the dichotomized outcome of the FMA. The 95 % CI show that the odds ratios are significant for components 2 (*p* = 0.014) and 3 (*p* = 0.003), but not for components 1 (*p* = 0.055) and 4 (*p* = 0.893).

**Table 3:** Multivariate logistic regression analysis

	Beta	Odds ratio	95 % CI	*p*
Component 1: Horizontal Shoulder Rotation–Elbow Flexion	−1.077	0.340	0.113–1.023	0.055
Component 2: Lateral Trunk Rotation–Upward Shoulder Rotation	1.484	4.409	1.352–14.382	0.014
Component 3: Forward Trunk Rotation–Axial Trunk Rotation–Elbow Flexion	−1.564	0.209	0.074–0.591	0.003
Component 4: External Shoulder Rotation–Forearm Pronation	−0.058	0.943	0.405–2.198	0.893

Dependent variable: FMA, dichotomized as 0 when FMA <34; 1 when FMA ≥34

## Discussion

To our knowledge, this is the largest cross-sectional study that uses PCA to investigate interactions between basic pathological synergies and compensatory motor control during a forward reach-to-grasp task with the paretic upper limb after stroke. With respect to the mean joint angles, we found significant differences in the use of Forward Trunk Rotation, Axial Trunk Rotation, Upward Shoulder Rotation, and Elbow Flexion, which indicates that the contribution of these degrees of freedom to reach-to-grasp is changed in patients with stroke relative to healthy individuals. PCA showed that most (84.7 %) of the variance in joint rotations of the trunk, shoulder, elbow, and lower arm during the reach-to-grasp task between patients with stroke could be explained by four components. Likewise, 86.6 % of the variance in reach-to-grasp could be explained by three components in healthy participants. The presence of the flexion synergy in patients with stroke, which can be observed as shoulder abduction combined with elbow flexion (Brunnstrom 1970) seemed to be reflected by component 1. Furthermore, component 2 suggests that Lateral Trunk Rotation is used to compensate for lack of shoulder contribution, whereas component 3 suggests that Forward and Axial Trunk Rotation are used to compensate for a lack of elbow movement. Component 4 explained primarily the variance in External Shoulder Rotation and Forearm Pronation, which implies that patients who use more External Shoulder Rotation also use more Forearm Pronation.

Apart from the contribution of Forward Trunk Rotation combined with Axial Trunk Rotation, all components that were identified in patients with stroke differed from the components identified in healthy individuals. This suggests that patients with stroke employ different functional linkages or synergies in order to execute this functional reach-to-grasp task. Moreover, the reflection of the flexion synergy in component 1 and the use of trunk movements to compensate for lack of shoulder (component 2) and elbow (component 3) contribution suggest that basic limb synergies and compensatory motor control play a crucial role during reach-to-grasp after stroke (Kwakkel et al. 2008; Levin et al. 2009).

Multivariate logistic regression analysis showed that in 38 of the 46 (82.6 %) included patients in the present study, the absence or presence of basic limb synergies could correctly be predicted by means of the component scores on the identified components. Specifically, the contribution of components 2 and 3 to the regression model was significant, which suggests that the use of compensatory trunk movements during reach-to-grasp is related to the presence of basic limb synergies as quantified by the FMA. The contribution of component 1, purportedly reflecting a flexion synergy, was not statistically significant; however, a trend between this component and the presence or absence of basic limb synergies could be discerned. These results support the hypothesis that basic limb synergies, as clinically determined, directly influence motor control strategies in patients with stroke. Moreover, the significant contribution of compensatory trunk movements (components 2 and 3) to the regression model is in line with a study by Subramanian et al. (2010) who also found a significant contribution of sagittal trunk displacement during reaching movements when using linear and logistic regression models to explain the variance in Fugl-Meyer motor assessment scores in 42 patients with stroke. Component 4, explaining the variance in Internal Shoulder Rotation and Forearm Pronation, did not contribute to the regression model, perhaps because these joint rotations are weaker contributors to basic limb synergies (Brunnstrom 1970; Twitchell 1951) and may therefore be harder to observe during the FMA.

The present results suggest that PCA is a promising tool to unravel the interaction between pathological upper limb synergies and compensatory movements of the trunk in patients with stroke. The current approach provides insight into the contribution of the relevant degrees of freedom in the paretic upper limb and trunk to the “sharing pattern” that is employed during the reach-to-grasp task. Latash states that this “sharing pattern” is the first feature of a functional synergy, whereas the second feature involves correction of spontaneous fluctuations in individual joint angles such that the performance variable (e.g., hand or finger position) remains unchanged. Using pointing movements, Reisman and Scholz (2003) did not find significant differences regarding this second feature between patients with stroke and healthy individuals, whereas the sharing pattern of patients with stroke could be captured by fewer principal components than in healthy individuals. This finding suggests that pointing movements in patients with stroke are more constrained. By contrast, the present study identified more principal components in patients with stroke, which could be explained by the fact that compensatory trunk movements were allowed in the present study, whereas Reisman and Scholz (2003) used a trunk restraint.

Despite the promising value of PCA to improve our insights into motor control after stroke, it remains unclear from the present study how impaired grasp function is related to the identified components. Reach-to-grasp was chosen as experimental paradigm since we consider the functionality of this task to be higher than reaching or pointing alone. Scores on the hand section of the Fugl-Meyer motor assessment were (sub)maximal for the patients in the present study (Table 1), suggesting that grasp impairments would have had minimal impact on the present results. Furthermore, it remains unclear to what extent the present results can be generalized to other functional tasks. Levin has shown that the correlation between shoulder and elbow rotations is larger in reaching movements to the ipsilateral side than in reaching movements to the contralateral side, suggesting that the presence of basic limb synergies depends on reaching direction (Levin 1996). In addition, the use of the trunk may vary substantially as objects are placed within or beyond reach (Levin et al. 2002). Hence, different task constraints may demand varying contributions of trunk, shoulder, and elbow rotations and could potentially lead to different component loadings in the PCA. More studies are therefore needed to investigate the effect of varying task constraints on functional synergies in patients with stroke.

The table height was fixed in the present study, which would have led to more shoulder abduction during the task in shorter individuals relative to taller individuals. Since the degree of shoulder abduction is known to be coupled to elbow flexion (Ellis et al. 2008), body length might have been a confounder for the detection of shoulder/elbow couplings (i.e., component 1), which might explain why the relation between component 1 and the presence or absence of basic limb synergies as quantified by the FMA could not significantly be established. Furthermore, it should be noted that a motion sensor on the pelvis was not incorporated during the 3D kinematic measurements, which may be seen as a limitation of the present study. Therefore, the reported trunk rotations in the present study should in fact be interpreted as combined trunk and pelvis rotations. However, in order to investigate interactions between compensatory movements and synergistic elbow and shoulder movements, we argue that it is not strictly relevant to know whether these compensatory movements are combined trunk and pelvis movements or trunk movements alone.

The variation between patients, and therefore the results of the PCA, may be affected not only by the specific experimental paradigm, but also by the accuracy of the 3D kinematic data and the reliability of the anatomical calibration. Previous experiments by our group showed, however, that the accuracy of the data is acceptable and constant over the entire measurement range (within 60 cm from the magnetic source) and the reliability of the anatomical calibration is high (van Kordelaar et al. 2012).

From this cross-sectional study, we conclude that PCA can be used to gain insight into the mutual relationships between motor impairments (reflected by basic limb synergies) and motor compensations (reflected by trunk movements) during functional movements with the paretic upper limb such as reach-to-grasp in patients with stroke. Insight into these relationships may help to optimize therapeutic approaches aimed at either restitution of motor control (i.e., reduction of basic limb synergies) early post stroke or compensatory motor control strategies that may be needed in later stages after stroke if restitution of motor control after stroke fails to occur (Langhorne et al. 2011). With that, PCA in longitudinal 3D kinematic studies may allow us to investigate *what* patients with stroke learn during skill acquisition in the first 6 months after stroke (Langhorne et al. 2011; Duncan et al. 1992; Kwakkel et al. 2006). Future studies with frequently repeated 3D kinematic measurements in time should therefore be used to investigate whether impairment-focused therapies, started in the first days post stroke, are able to restore motor control by “true” neurological repair beyond mechanisms of spontaneous neurological recovery (Kwakkel et al. 2008).

## Appendix

The calculations of the rotations of the trunk, shoulder, and elbow were adopted from the recommendations of the ISB (Wu et al. 2005). These calculations consisted of two parts: (1) the construction of the segment reference frames of the trunk, upper arm, and forearm and (2) the decomposition of trunk, shoulder, and elbow orientations into orthogonal rotations.

### Construction of segment reference frames

By using a stylus (ST8, Polhemus), the positions of 13 anatomical landmarks (Table 4) were digitized prior to each measurement with respect to the global reference frame of the electromagnetic motion tracker (Polhemus Liberty, Polhemus, Vermont, USA). In addition, the location of the gleno-humeral joint was calculated using linear regression from the scapular landmarks (Meskers et al. 1998). Subsequently, each landmark position was rotated from the global reference frame into the local reference frame of its associated sensor:

1 $$ P_{{{\text{lm}}_{i} }}^{\text{sens}} = {\text{inv}}\left( {T_{\text{sens}}^{\text{glo}} } \right) \times P_{{{\text{lm}}_{i} }}^{\text{glo}},$$

where $P_{{{\text{lm}}_{i} }}^{\text{sens}}$ is a vector representing the position of landmark i with respect to the reference frame of its sensor, $T_{\text{sens}}^{\text{glo}}$ is a transformation matrix representing the sensor reference frame with respect to the global reference frame, and $P_{{{\text{lm}}_{i} }}^{\text{glo}}$ is a vector representing the digitized position of landmark i with respect to the global reference frame. Segment reference frames for the trunk, upper arm, and forearm were defined on the basis of the positions of the anatomical landmarks with respect to the sensor reference frame and according to the recommendations of the ISB (Wu et al. 2005). We used the first option of the ISB recommendations for the definition of upper arm reference frame. The segment reference frames were fixed with respect to the sensor reference frames and, in the anatomical position, the longitudinal, the transversal, and the sagittal axes of these segment reference frames pointed upward, rightward, and forward, respectively. The three-dimensional positions and orientations of the trunk, upper arm, and forearm during the motion recordings, that is, the transformation matrices of each segment reference frame at each time sample (*t*), were derived by multiplying the measured sensor transformation matrix at each time sample (*t*) by the transformation matrix of the segment reference frame with respect to its associated sensor.

2 $$ T_{\text{seg}}^{\text{glo}} (t) = T_{\text{sens}}^{\text{glo}} (t) \times T_{\text{seg}}^{\text{sens}} , $$

where $T_{\text{seg}}^{\text{glo}}$ represents the transformation matrix describing the position and orientation of the segment reference frame relative to the global reference frame, $T_{\text{sens}}^{\text{glo}}$ represents the transformation matrix describing the position and the orientation matrix of the sensor relative to the global reference frame, and $T_{\text{seg}}^{\text{sens}}$ represents the transformation matrix describing the fixed position and the orientation matrix of the segment reference frame relative to its associated sensor, which was determined in Eq. 1.

**Table 4:** Anatomical landmarks

Anatomical landmarks (defined in anatomical position)	Description
Thorax	
IJ: incissura jugularis	Deepest point (suprasternal notch)
PX: processus xiphoideus	Most caudal point on the sternum
C7: processus spinosus 7th cervical vertebra	Most dorsal point
T8: processus spinosus 8th thoracic vertebra	Most dorsal point
Scapula	
TS: trigonum spinae	Midpoint of the triangular surface on the medial border of the scapula in line with the scapular spine
AI: angulus inferior	Most caudal point of the scapula
AA: angulus acromialis	Most laterodorsal point of the scapula
PC: processus coracoideus	Most ventral point of the scapula
AC: acromio-clavicular joint	Most dorsal point of the acromio-clavicular joint
Humerus	
GH: gleno-humeral rotation center^a^	Rotation center of the gleno-humeral joint
EL: lateral epicondyle	Most caudal point on the EL
EM: medial epicondyle	Most caudal point on the EM
Lower arm	
US: ulnar styloid	Most caudal and medial point on the US
RS: radial styloid	Most caudal and lateral point on the RS

^a^Determined by means of linear regression from the scapular landmarks (Meskers et al. 1998)

### Decomposition of trunk, shoulder, and elbow orientations

From the 4 × 4 transformation matrices, specifying the position and orientation of each segment relative to the global reference frame ($T_{\text{seg}}^{\text{glo}}$), the 3 × 3 orientations matrices were used for further analyses ($O_{\text{seg}}^{\text{glo}}$). Trunk orientation at each time sample (*t*) was described as the orientation of the trunk reference frame with respect to the global reference frame: $O_{\text{trunk}}^{\text{glo}}$.

Shoulder orientation at each time sample (*t*) was described as the orientation of the upper arm reference frame with respect to the trunk reference frame

3 $$ O_{\text{upperarm}}^{\text{trunk}} (t) = {\text{inv}}(O_{\text{trunk}}^{\text{glo}} (t)) \times O_{\text{upperarm}}^{\text{glo}} (t), $$

where $O_{\text{upperarm}}^{\text{trunk}}$ represents the orientation of the upper arm relative to the trunk, $O_{\text{upperarm}}^{\text{glo}}$ represents the orientation of the upper arm relative to the global reference frame, and $O_{\text{trunk}}^{\text{glo}}$ represents the orientation of the trunk relative to the global reference frame.

Elbow orientation at each time sample (*t*) was described as the orientation of the forearm reference frame with respect to the upper arm reference frame.

4 $$ O_{\text{forearm}}^{\text{upperarm}} (t) = {\text{inv}}(O_{\text{upperarm}}^{\text{glo}} (t)) \times O_{\text{forearm}}^{\text{glo}} (t) $$

where $O_{\text{forearm}}^{\text{upperarm}}$ represents the orientation of the forearm relative to the upper arm, $O_{\text{upperarm}}^{\text{glo}}$ represents the orientation of the upper arm relative to the global reference frame, and $O_{\text{forearm}}^{\text{glo}}$ represents the orientation of the forearm relative to the global reference frame.

Subsequently, the three-dimensional rotations of the trunk, shoulder, and elbow were obtained by decomposing their orientation matrices into orthogonal rotations in the following orders:

Trunk rotations

- Forward/Backward Trunk Rotation: Rotation of the trunk reference frame about the transversal axis of the global reference frame (0°: trunk upright; positive value: forward rotation; negative value: backward rotation).
- Lateral Trunk Rotation: Rotation of the trunk reference frame about its sagittal axis (0°: trunk upright; positive value: rotation toward the measured side; negative value: rotation away from the measured side).
- Axial Trunk Rotation: Rotation of the trunk reference frame about its longitudinal axis (0°: neutral position; positive value: rotation away from the measured side; negative value: rotation toward the measured side)

Shoulder rotations

- Horizontal Shoulder Rotation: Rotation of the upper arm about the longitudinal axis of the trunk (0°: abduction; 90°: anteflexion).
- Upward Shoulder Rotation: Angle between the longitudinal axis of the trunk and the longitudinal axis of the upper arm (0°: longitudinal axes of the upper arm and trunk perfectly aligned; positive value: upward rotation of the upper arm).
- Internal/External Shoulder Rotation: Rotation of the upper arm about its longitudinal axis (0°: neutral position; positive value: internal rotation; negative value: external rotation).
- A detailed description of these shoulder angles is provided by Doorenbosch and colleagues (Doorenbosch et al. 2003).

Elbow rotations

- Elbow Flexion: Rotation of the forearm about the axis through the medial and lateral epicondyles of the upper arm (0°: fully extended; positive angle: flexion).
- Elbow Adduction/Abduction: This rotation cannot be performed by the human elbow and was therefore omitted in the present study.
- Forearm Pronation/Supination: Rotation of the forearm about its longitudinal axis (0°: fully supinated; positive value: pronation).

The data analysis was conducted using an adapted version of BodyMech 3.06.01 (van Andel et al. 2008; Doorenbosch et al. 2003).
